# Differential basolateral–apical distribution of scavenger receptor, class B, type I in cultured cells and the liver

**DOI:** 10.1007/s00418-014-1251-9

**Published:** 2014-07-25

**Authors:** Stefanie Fruhwürth, Werner J. Kovacs, Robert Bittman, Simon Messner, Clemens Röhrl, Herbert Stangl

**Affiliations:** 1 Center for Pathobiochemistry and Genetics, Department of Medical Chemistry, Medical University of Vienna, Währingerstraße 10, 1090 Vienna, Austria; 2 Institute of Molecular Health Sciences, Swiss Federal Institute of Technology Zurich (ETHZ), Zurich, Switzerland; 3 Department of Chemistry and Biochemistry, Queens College of the City University of New York, Flushing, NY USA; 4 InSphero AG, Schlieren, Switzerland

**Keywords:** SR-BI, Cholesterol, HDL, Canalicular membranes, HepG2, BODIPY-cholesterol

## Abstract

The high-density lipoprotein (HDL) receptor, scavenger receptor class B, type I (SR-BI), mediates selective cholesteryl ester uptake into the liver, which finally results in cholesterol secretion into the bile. Despite several reports, the distribution of hepatic SR-BI between the sinusoidal and canalicular membranes is still under debate. We present immunohistological data using specific markers showing that the bulk of SR-BI is present in sinusoidal membranes and, to a lesser extent, in canalicular membranes in murine and human liver sections. In addition, SR-BI was detected in preparations of rat liver canalicular membranes. We also compared the in vivo findings to HepG2 cells, a widely used in vitro hepatocyte model. Interestingly, SR-BI was enriched in bile canalicular-like (BC-like) structures in polarized HepG2 cells, which were cultivated either conventionally to form a monolayer or in Matrigel to form three-dimensional structures. Fluorescently labeled HDL was transported into close proximity of BC-like structures, whereas HDL labeled with the fluorescent cholesterol analog BODIPY-cholesterol was clearly detected within these structures. Importantly, similarly to human and mouse liver, SR-BI was localized in basolateral membranes in three-dimensional liver microtissues from primary human liver cells. Our results demonstrate that SR-BI is highly enriched in sinusoidal membranes and is also found in canalicular membranes. There was no significant basolateral–apical redistribution of hepatic SR-BI in fasting and refeeding experiments in mice. Furthermore, in vitro studies in polarized HepG2 cells showed explicit differences as SR-BI was highly enriched in BC-like structures. These structures are, however, functional and accumulated HDL-derived cholesterol. Thus, biological relevant model systems should be employed when investigating SR-BI distribution in vitro.

**Electronic supplementary material:**

The online version of this article (doi:10.1007/s00418-014-1251-9) contains supplementary material, which is available to authorized users.

## Introduction

The scavenger receptor class B, type I (SR-BI), a cell surface glycoprotein that binds high-density lipoprotein (HDL), low-density lipoprotein (LDL), very low-density lipoprotein (VLDL), modified LDL, and anionic phospholipid (Acton et al. 1994, 1996; Rigotti et al. 1995; Calvo et al. 1997), mediates the last step in reverse cholesterol transport, namely the delivery of cholesteryl esters and other lipids from HDL to liver cells for disposal into the bile. This process, in which HDL- and LDL-derived cholesteryl esters are transferred to the cell without the concomitant degradation of the lipoprotein particle, is termed selective cholesteryl ester uptake (Acton et al. 1996; Glass et al. 1983, 1985; Stangl et al. 1998, 1999; Swarnakar et al. 1998). Of all organs, SR-BI expression is most abundant in the liver (Krieger 1999). Consequently, the liver accounts for up to 90 % of selective HDL cholesteryl ester uptake in rats and mice (Glass et al. 1983, 1985; Stein et al. 1983; Pittman and Steinberg 1984; Knecht and Pittman 1989). As expected, less selective cholesteryl ester uptake from HDL particles (~20 %) is found in the liver of cholesteryl ester transfer protein (CETP)-expressing animals (Goldberg et al. 1991). Hepatic SR-BI expression is regulated by dietary, hormonal, and pharmacological interventions; for reviews see (Kent and Stylianou 2011) (Landschulz et al. 1996; Fluiter et al. 1998; Serougne et al. 1999).

There is a puzzling discrepancy about the reports on the localization of hepatic SR-BI: Under basal conditions, most hepatic SR-BI resides on parenchymal cells (Fluiter et al. 1998; Stangl et al. 2002; Mardones et al. 2003) and is most exclusively located on the sinusoidal surface of hepatocytes (Mardones et al. 2003; Stangl et al. 2002). However, in mice overexpressing SR-BI, SR-BI was detected in the canalicular domains of hepatocytes by immunohistochemistry (Kozarsky et al. 1997; Ikemoto et al. 2000; Sehayek et al. 2003). SR-BI was also detected on cultured hepatocyte couplets using WIF-B9 cells (Silver et al. 2001). In addition to its expression in liver parenchymal cells, SR-BI protein has been detected in Kupffer (Fluiter et al. 1998; Hoekstra et al. 2005; Malerod et al. 2002) and liver endothelial cells (Hoekstra et al. 2005; Malerod et al. 2002). Interestingly, an atherogenic diet was shown to induce SR-BI expression several fold in Kupffer cells (Fluiter et al. 1998; Hoekstra et al. 2005) and to a much lesser extent in liver parenchymal and endothelial cells in mice. However, the precise role of SR-BI in Kupffer cells remains to be elucidated.

Several lines of evidence demonstrate that SR-BI is redistributed between apical and basolateral membranes as a function of their cholesterol levels. For example, Harder et al. (2007) showed that SR-BI undergoes transcytosis to apical bile canalicular-like (BC-like) structures upon cholesterol loading in polarized hepatocytes (WIF-B cells). Conversely, transcytosis of SR-BI was observed upon cholesterol depletion in MDCK cells (Burgos et al. 2004). These data indicate regulated apical–basolateral cell surface protein distribution in cell models.

Since the liver is characterized by a rather complex polarity, the question still remains how SR-BI is distributed between sinusoidal and canalicular membranes. To clarify this, we investigated the localization of SR-BI protein in human and rodent liver sections. We employed immunohistological, biochemical, and cell biological methods to show the expression of SR-BI in sinusoidal membranes and to a lesser extent in canalicular membranes.

## Materials and methods

### Antibodies

The following antibodies were used: anti-MDR1 (ABCB1; Kamiya Biomedical Company, USA) 1:200; anti-ABCB11 (BSEP; Santa Cruz, USA) 1:50; anti-MRP2 (ABCC2; Enzo Life Sciences, USA) 1:50; anti-β-catenin (Life Technologies, USA) 1:500; anti-SR-BI (CLA-1; BD Biosciences, USA) 1:50 for HepG2 cells; anti-SRBI (Stangl et al. 1998) 1:200 for mouse liver and HepG2, Huh7 and human liver microtissues; anti-SRBI (kindly provided by A. Ritsch, Med. Univ. Innsbruck, Austria) 1:200 for human liver; anti-LAP (kindly provided by A. Rigotti, Universidad Católica de Chile, Santiago, Chile); anti-LDLR (kindly provided by M.S. Brown and J.L. Goldstein, UTSW, Dallas, TX).

### Cell culture

HepG2 cells were maintained in minimal essential medium (MEM, GE Healthcare, UK) supplemented with 10 % fetal bovine serum (FBS; Gibco, Life Technologies), 1 % nonessential amino acid solution (Sigma, USA), 1 mM sodium pyruvate (PAA, Austria), and penicillin/streptomycin (PAA). For microscopy on HepG2 monolayers, cells were grown on coverslips in 24-well plates at a density of 2 × 10^5^ cells/well.

### HepG2 spheroid formation and harvesting

A single cell suspension of HepG2 cells (~6 × 10^3^ cells/ml) in cold medium was mixed 1:3 with Matrigel (Basement Membrane Matrix Growth Factor Reduced; BD Biosciences). From this suspension, aliquots of 50 µl were placed into the middle of 24 wells. Plates were incubated at 37 °C for 30 min until the Matrigel was gelled. Then, warm medium was added. On day 4, HepG2 spheroids were harvested using a cell recovery solution (Corning, USA) according to the manufacturer’s protocol.

### Lipoprotein isolation and labeling with fluorescent dyes

Plasma was collected from healthy volunteers, and HDL was prepared by sequential ultracentrifugation (*d* = 1.21 g/ml) (Schumaker and Puppione 1986). The apolipoprotein part of HDL was covalently labeled with Alexa Fluor 488 (Molecular Probes, USA) according to the manufacturer’s instructions. Loading of HDL with BODIPY-cholesterol (BP-C) (Li et al. 2006) was performed as described previously (Rohrl et al. 2012). HepG2 cells were incubated with 50 µg/ml of labeled HDL for the indicated time points. Thereafter, cells were fixed in 4 % formaldehyde at 4 °C for 30 min, and samples were mounted and examined using a confocal microscope (LSM 5 Exciter, Zeiss, Germany).

### TRITC-phalloidin staining

The TRITC-phalloidin (Sigma) stock solution of 0.1 mg/ml in DMSO was diluted 1:500 in PBS. After fixation, HepG2 cells were washed twice with PBS, incubated with the phalloidin working solution for 20 min at room temperature, and washed twice with PBS.

### Immunofluorescence on HepG2 monolayers and spheroids

HepG2 cells were fixed in 4 % formaldehyde at 4 °C for 30 min. Samples were then washed twice with PBS and blocked with PBS containing 2 % BSA (Sigma) and 0.05 % saponin (Sigma) at room temperature for 1 h, followed by incubation with primary antibodies diluted in PBS containing 1 % BSA and 0.05 % saponin (buffer A). After harvesting and fixation in 4 % formaldehyde at 4 °C for 30 min, HepG2 spheroids were blocked with PBS containing 5 % BSA and 1 % Triton-X100 (Sigma) (buffer B) at room temperature for 1 h and incubated with primary antibodies diluted in buffer B overnight at 4 °C. Next, cells were washed twice with PBS and incubated with secondary antibodies diluted in buffer A (HepG2 monolayers) or buffer B (HepG2 spheroids) at room temperature for 1 h. Thereafter, cells were washed twice with PBS, and phalloidin staining and counter staining using DAPI were performed. The samples were mounted with Fluoprep (Biomerieux, France) and imaged using a fluorescence microscope (Axiovert 135, Zeiss) or a confocal microscope (LSM 5 Exciter).

### HepG2 and Huh7 liver microtissue production and culture

Paraffin sections from 3D InSight™ HepG2 and Huh7 liver microtissues were obtained from InSphero AG (Schlieren, Switzerland). The cells were seeded in hanging-drop plates (GravityPLUS™) for microtissue re-aggregation and further cultivated in GravityTRAP™ plates in 3D InSight™ HepG2 Liver Maintenance Medium (InSphero AG). The tissues were cultivated for 14 days before formalin fixation and paraffin embedding. Paraffin sections were processed similarly as mouse liver sections.

### Human liver microtissue production and culture

Paraffin sections from 3D InSight™ Human Liver Microtissues were obtained from InSphero AG. The liver microtissues consisted of primary human hepatocytes (lot IZT) in co-culture with primary non-parenchymal cells (lot JJB). The mixture of primary human liver cells were seeded in hanging-drop plates (GravityPLUS™) for microtissue re-aggregation and further cultivated in GravityTRAP™ plates in 3D InSight™ Human Liver Maintenance Medium (InSphero AG). The tissues were cultivated for the indicated time points and harvested for histology. Formalin-fixed microtissues were paraffin embedded, sectioned, and processed similarly as mouse liver sections.

### Immunofluorescence on human and mouse liver sections

Normal human liver sections were obtained from US Biomax (USA). Mouse liver samples were retrieved from 11-week-old male C57BL/6 J mice. All protocols for animal use and experiments were reviewed and approved by the Austrian Ministry of Science and Research. Mice were kept on chow under standard conditions and had free access to food and water. After transcardial perfusion with PBS, the liver was collected, fixed in 4.5 % formaldehyde for 24 h, and afterward paraffin embedded. Human and mouse liver sections were processed similarly, and deparaffinization and antigen retrieval were conducted according to standard procedures. Afterward, liver sections were washed twice with TBS, blocked in TBS containing 5 % BSA (buffer C) at room temperature for 1 h, followed by incubation with primary antibodies diluted in buffer C at 4 °C overnight. Samples were washed twice with TBS and incubated with secondary antibodies diluted in buffer C at room temperature for 1 h. The procedure was adapted for the primary mouse anti-MRP2 and mouse anti-β-catenin antibodies on mouse tissue using the M.O.M.-Kit (Vector Laboratories, USA) according to the manufacturer’s instructions. Finally, samples were washed twice with TBS, DAPI stained, washed twice with TBS, and mounted using Fluoprep. Liver sections were imaged using a confocal microscope (LSM 5 Exciter). Images of the fasting–refeeding experiment were taken with a Leica SP2-AOBS confocal laser scanning microscope. Fluorescent dyes were imaged sequentially in frame interlace mode to eliminate cross talk between the channels.

### Fasting and refeeding experiments

Ten-week-old male C57BL/6 J mice were obtained from Elevage Janvier (France) and were acclimatized to local animal facility conditions for two weeks prior to the fasting and refeeding experiment. Mice were housed at 22 °C with a 12/12-h light/dark cycle (lights on at 6 a.m. and off at 6 p.m.) and provided water and standard rodent chow diet ad libitum. Mice were randomly assigned to the experimental groups. For the fasting and refeeding experiment, mice were either used in the ad libitum fed state or fasted for 24 h, or fasted for 24 h and subsequently refed with a standard chow diet for 12 h. Mice were killed at 9 a.m. Mice were anesthetized with ketamine hydrochloride and xylazine, and the liver was perfused with PBS for 2 min and subsequently with 4 % formaldehyde. The fasting–refeeding experiments were reviewed and approved by the Veterinary Office of Zürich (Switzerland).

### Preparation of rat liver canalicular plasma membranes

Canalicular membranes were prepared according to Inoue et al. (1983a, b): Aliquots of the intermediate steps were kept to monitor the distribution of the proteins and enzyme activities and estimate their enrichment or depletion. Male Sprague–Dawley rats, 200–250 g, were kept on standard chow and housed at a light–darkness cycle of 12 h in the animal facility (Stangl et al. 2002). Rats were anesthetized, and their livers were perfused in situ with 30 ml of ice-cold saline and subsequently with 20 ml of 0.25 M sucrose, 10 mM Hepes-Tris buffer, pH 7.4, and 0.2 mM CaCl_2_ (medium A). The liver was excised, minced, and homogenized with 30 strokes in medium A (60 ml/12.5 g liver weight) using a loose fitting Dounce homogenizer (Sigma) at 0 °C. After filtration through cheesecloth (Aliquot S1), the homogenate was diluted to 160 ml with ice-cold medium A with 1 mM EDTA. After centrifugation for 10 min at 1,800*g*, the pellet and fluffy layer were collected and resuspended in 80 ml of medium A (Aliquot S2). The sample was re-centrifuged at 3,000*g* for 10 min. The pellet was suspended in 30 ml of medium A, placed in a high-pressure chamber (Parr Instrument, USA), and equilibrated with nitrogen at 800 psi for 15 min with gentle stirring at 0 °C. Then, the pressure was suddenly released and 95 ml of ice-cold medium A containing 14 mM CaCl_2_ was added to the homogenate, and the mixture was stirred gently for 10 min (Aliquot SA). After centrifugation for 20 min at 7,600*g*, the supernatant was collected (Aliquot SB) and centrifuged for 20 min at 47,000*g* (Aliquot SC). The resulting pellet was resuspended in 8 ml of ice-cold medium A and again centrifuged at 47,000*g* for 30 min (Aliquot CM). The resulting pellet was resuspended in about 200 µl of medium A using a syringe and needle (26 gauge) and afterward stored at −80 °C for Western blot analysis. Enzymatic analysis of 5′-nucleosidase and acid and alkaline phosphatase was performed using freshly prepared samples using standard test kits from Sigma.

## Results

### SR-BI is localized to BC-like structures in human hepatoma cell lines

**Fig. 1:**
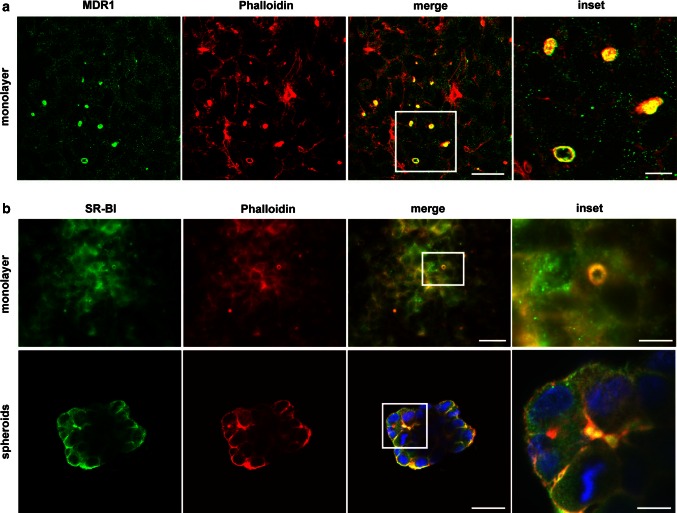
SR-BI localization in vitro and delivery of HDL-derived cholesterol to BC-like structures. **a** HepG2 cells were either cultivated under standard conditions to form a monolayer (*upper panel*) or allowed to form spheroids (*lower panel*). Immunofluorescence staining for SR-BI was performed, followed by phalloidin staining. Spheroids were imaged using a confocal microscope; monolayers were imaged using a conventional fluorescence microscope. *Bar* = 40 µm; bar for inset = 10 µm. *Blue* = DAPI. **b** HepG2 cells were incubated with 50 µg/ml of HDL-Alexa 488 (*upper panel*) or HDL-BODIPY-cholesterol (HDL-BP-C; *lower panel*) for the indicated time points. Afterward, cells were fixed, and phalloidin staining was performed. Cells were imaged using a confocal microscope. *Bar* = 25 µm; for inset *bar* = 6 µm

Primary hepatocytes lose their polarity within hours (Tsukamoto et al. 2013; Graf et al. 1984), but some hepatocyte couplets form BC-like structures (Graf et al. 1984). Similarly, human hepatoma cell lines are capable of forming BC-like structures (Sormunen et al. 1993; Chiu et al. 1990). In HepG2 cells, such BC-like structures, which are surrounded by actin filaments as shown by phalloidin staining, were detected (Supplementary Fig. 1). These BC-like structures exhibited a strong co-localization of phalloidin and multidrug resistance protein 1 (MDR1), an efflux transporter localized to the apical canalicular membrane in the liver. Thus, phalloidin staining is an appropriate marker of these structures in this in vitro model and was used to identify BC-like formations. SR-BI was enriched at the apical side of HepG2 cells (Fig. 1a, upper panel) and co-localization with phalloidin was regularly detected (see insert), indicating that SR-BI is in part localized at BC-like structures in HepG2 cells.

We next sought to compare the HepG2 monolayer culture with a more physiological three-dimensional (3D) model. Therefore, HepG2 cells were cultivated in Matrigel and allowed to form spheroids (Fig. 1a, lower panel). In HepG2 monolayers and spheroids, SR-BI was detected at the cell membrane and co-localized with phalloidin, indicating that SR-BI is localized to BC-like structures in HepG2 cells independent of the culturing conditions. Next, we compared the localization of SR-BI in HepG2 and Huh7 microtissues with 3D human liver microtissues, all of which were established using the hanging-drop culture (Supplementary Fig. 2). SR-BI was localized to the plasma membrane in HepG2 (Supplementary Fig. 2a) and Huh7 (Supplementary Fig. 2b) microtissues with enrichment in BC-like structures (compare with Fig. 1). 3D human liver microtissues generated from primary human parenchymal cells (hepatocytes) and liver-derived non-parenchymal cells have been shown to retain long-term viability and functionality in culture (Messner et al. 2013). Immunofluorescence analysis revealed that SR-BI is localized to the plasma membrane in 3D human liver microtissues (Supplementary Fig. 2c). SR-BI distribution did not differ between microtissues that were cultured for 7, 14, 21, or 28 days. Our data, along with previously published data showing the distribution of apical markers in liver microtissues (Messner et al. 2013), suggest that SR-BI is localized in the basolateral membrane of primary human liver microtissues, closely mimicking the in vivo situation.

### HDL-derived lipids are delivered to BC-like structures

We next assessed the functionality of BC-like structures in HepG2 cells with regard to their handling of HDL-derived lipids. Therefore, HDL was labeled with either Alexa 488 at the apolipoprotein moiety (HDL-Alexa 488) or the fluorescent cholesterol analog BODIPY-cholesterol (HDL-BP-C) (Li et al. 2006). Alexa 488-labeled HDL was found in close proximity to BC-like structures, but almost no co-localization with phalloidin was observed (Fig. 1b). This indicates that HDL is endocytosed, comes in close contact with the apical membrane, and possibly transfers lipids to the apical membrane. In contrast, HDL-BP-C was found within BC-like structures after only 15 min, showing that HDL-derived cholesterol is rapidly transported to the apical membrane (for serial z-stack images, see Supplementary Fig. 3); similar findings were reported previously (Wüstner et al. 2004). Thus, our results indicate that the BC-like structures formed by HepG2 cells are functional in translocating cholesterol as HDL-BP-C was clearly detected within these structures.

### SR-BI is mainly localized to the sinusoidal membrane in rodent liver

**Fig. 2:**
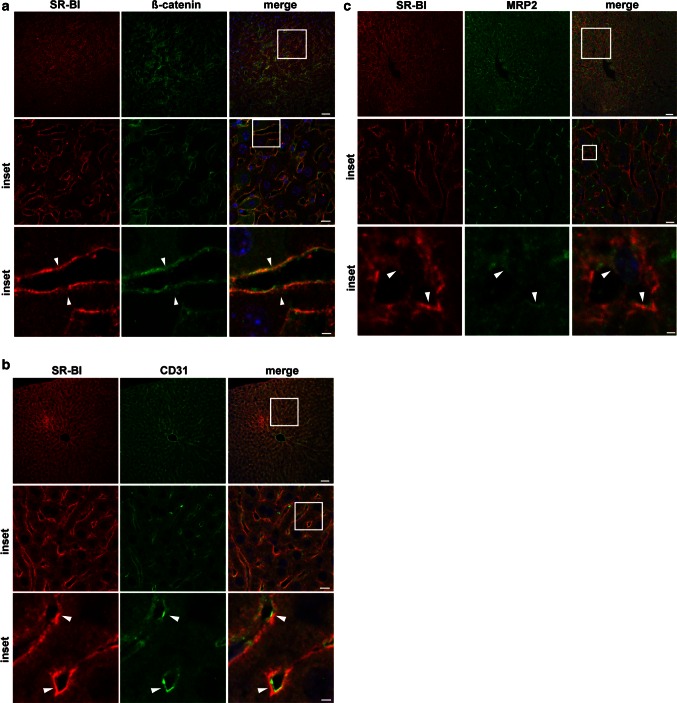
Immunohistological analysis of SR-BI localization in vivo in mouse liver. **a**–**c** mouse liver sections were stained for SR-BI (*red*) and either β-catenin (*green* **a**), or CD31 (*green* **b**), or MRP2 (*green* **c**) and imaged using a confocal microscope. SR-BI was found beneath endothelial cells (*arrowheads* in **b**) and to co-localize with β-catenin (*arrowheads* in **a**) and to some extent with MRP2 (*arrowheads* in **c**). *Bar* = 50 µm; for *middle panel* *bar* = 10 µm, for *lower panel* *bar* = 3 µm (**a**, **b**) and 1.5 µm (**c**). *Blue* = DAPI

We further investigated the localization of SR-BI in vivo in murine liver. β-Catenin, which plays an important role in the formation of adherent junctions, was used as a marker for the sinusoidal membrane of hepatocytes. SR-BI co-localized strongly with β-catenin (Fig. 2a, arrowheads). In addition, intense SR-BI staining was detected adjacent to sinusoidal endothelial cells, which were stained with the endothelial cells marker CD31 (Fig. 2b, arrowheads). Only a minor amount of SR-BI was expressed at the canalicular membrane, as shown by co-staining with the canalicular transporter multidrug resistance-associated protein 2 (MRP2) (Fig. 2c, arrowheads).

**Fig. 3:**
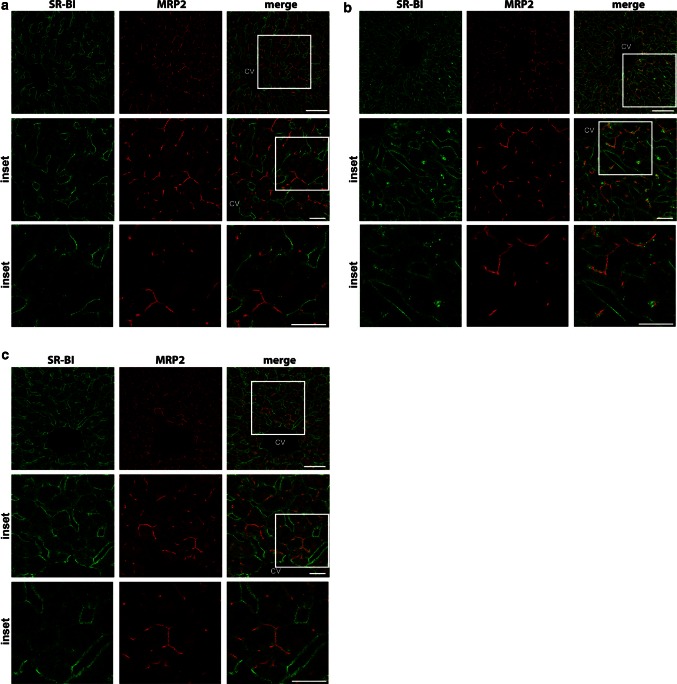
Immunohistological analysis of SR-BI localization upon fasting and refeeding. **a**–**c**, immunofluorescence analysis of SR-BI (*green*) and MRP2 (*red*) in livers from C57BL/6 J mice fed ad libitum (**a**), fasted for 24 h (**b**), and fasted for 24 h and the refed for 12 h (**c**) using confocal microscopy. *Bar* = 50 µm for *upper panels*. *Bar* = 20 µm for* middle* and *lower panels*. *CV* central vein

Next, we examined whether fasting and refeeding affect the localization of SR-BI in mouse liver (Fig. 3). Fasting is a powerful stimulator of adipose tissue lipolysis, which gives rise to a profound increase in plasma fatty acid content. These fatty acids are readily taken up by the liver and lead to a transient hepatic steatosis, which regresses rapidly after refeeding. Marked SR-BI immunostaining was observed in the membranes facing the sinusoids in livers from ad libitum fed (Fig. 3a), 24-h fasted (Fig. 3b), and 24-h fasted/12-h refed mice (Fig. 3c), whereas only minor staining was present in the canalicular membranes of the hepatocytes. Interestingly, there was an increased spotty cellular SR-BI immunoreactivity in the proximity of MRP2-positive canalicular membranes in hepatocytes of mice fasted for 24 h (Fig. 3b).

**Fig. 4:**
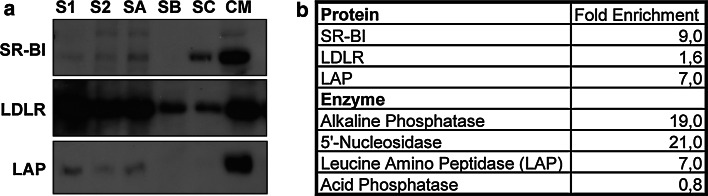
Biochemical investigation of SR-BI localization in vivo in rat liver. **a** Western blot analysis of starting material, intermediate aliquots and final canalicular membranes is shown. *S1* supernatant 1; *S2* supernatant 2; *SA* supernatant A; *SB* supernatant B; *SC* supernatant C; *CM* canalicular membrane. **b** Enrichment of corresponding proteins was assessed by densitometric calculation (protein) and measured enzymatic activity (enzyme). Note the enrichment of SR-BI in membrane fractions of rat liver representing canalicular membranes

To further substantiate the immunofluorescence data, we prepared canalicular membranes from rat liver. SR-BI protein levels were enriched in these membranes over starting material S1 (ninefold), while the LDL receptor (LDLR), known to cycle between endosomal compartments and the sinusoidal membrane, was not specifically enriched (1.6-fold) (Fig. 4). The canalicular marker leucine amino peptidase was increased about sevenfold in this membrane preparation using both Western blot analysis and enzymatic measurements. The enzymatic activities of alkaline phosphatase and 5′-nucleosidase, two known canalicular marker proteins, were increased by 19- and 21-fold, respectively. In contrast, the endosomal/lysosomal marker acid phosphatase was depleted in this membrane fraction by 0.8-fold, based on enzymatic measurements (Fig. 4b). Thus, our data demonstrate that canalicular membranes contain detectable amounts of SR-BI protein; however, SR-BI is not as highly expressed as classical proteins residing in the canalicular membrane such as 5′-nucleosidase and alkaline phosphatase.

### SR-BI is primarily found at the sinusoidal membrane in human liver

**Fig. 5:**
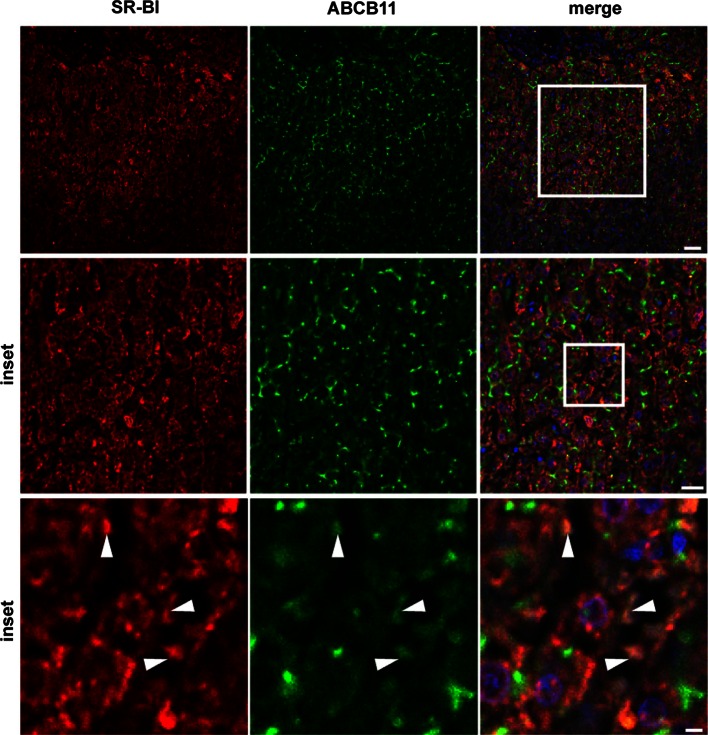
Immunohistological analysis of SR-BI localization in vivo in human liver. Human liver sections were stained for SR-BI (*red*) and ABCB11 (*green*) and imaged using a confocal microscope. Partial co-localization of SR-BI and ABCB11 was found (*arrowheads*). *Bar* = 50 µm; for* middle panel bar* = 30 µm, for *lower panel* *bar* = 6 µm. *Blue* = DAPI

Finally, we investigated the localization of SR-BI in human liver (Fig. 5). The bile salt export pump ATP-binding cassette transporter B11 (ABCB11) was used as a marker of the apical membrane of hepatocytes. The majority of SR-BI was found on the membrane facing the sinusoids, whereas only a minor amount was detected at the canalicular membrane, as shown by co-localization with ABCB11 (see arrowheads).

## Discussion

In this study, we show with immunohistological and biochemical methods that SR-BI is mainly expressed on sinusoidal membranes in the liver of rodents and humans. Nevertheless, SR-BI is also detected at canalicular membranes, although to a much lesser extent. Interestingly, we observed a strong SR-BI staining at the membrane of BC-like structures in the human hepatoma cell lines HepG2 and Huh7, indicating that this cell models differ substantially from the in vivo situation. Furthermore, HDL-derived cholesterol was found within these BC-like structures in HepG2 cells. Microtissues, derived from primary human hepatocytes, maintain a SR-BI distribution pattern similar to that found in liver even when they were cultivated for up to 28 days.

Even though polarized hepatic cell lines were shown to mimic some of the liver-specific properties and functions such as bile canaliculi formation (Sormunen et al. 1993; Chiu et al. 1990), especially when the cells are cultivated in 3D structures (for a review see: Decaens et al. 2008), they display considerable differences (Tsukamoto et al. 2013). We show that there is a significant discrepancy in the distribution of SR-BI, which was enriched at the apical membrane in cultured HepG2 and Huh7 cells, whereas in vivo only a minor amount was associated with canalicular membranes. Interestingly, human 3D liver microtissues displayed a SR-BI distribution pattern that was very similar to the in vivo situation in human and murine liver. Based on a previous careful characterization of these liver microtissues (Messner et al. 2013), we conclude that, like in the liver, SR-BI is mainly localized in basolateral membranes and only a minor amount is found in the apical membranes or non-parenchymal cells such as Kupffer cells.

HDL-derived BODIPY-cholesterol was efficiently transported into the BC-like, MDR1 expressing structures formed by HepG2 cells, whereas Alexa-labeled HDL did not reach these structures. This is in agreement with previous reports showing selective sorting of HDL-derived lipids and the HDL particle in hepatic cell lines (Wüstner 2005; Wüstner et al. 2004).

We could clearly demonstrate in vivo that SR-BI is associated with canalicular membranes in mice, rats, and humans using different experimental approaches. In addition to immunohistological analyses of livers from chow-fed mice and healthy humans, we prepared liver canalicular membranes from chow-fed rats. SR-BI protein was concomitantly enriched with the canalicular marker leucine amino peptidase in the canalicular membrane fraction, indicating that some SR-BI is partitioned in bile canalicular membranes. In contrast, the LDLR, which cycles between endosomes and the sinusoidal membrane, served as a negative control. Accordingly, LDLR protein was highly abundant in the starting material and gradually decreased during the canalicular membrane purification with almost no enrichment in the final fraction.

The interaction of SR-BI with proteins containing a PDZ domain is crucial for its location (Ikemoto et al. 2000; Tsukamoto et al. 2013; Hu et al. 2013). PDZ domain-containing protein 1 (PDZK1) is the major determinant of SR-BI expression and localization in the liver, which was shown to be associated with SR-BI residing in sinusoidal membranes (Ikemoto et al. 2000). In PDZK1 knockout mice, hepatic SR-BI is dramatically reduced and redistributed from the cell surface of hepatocytes to intracellular regions (Fenske et al. 2008). The regulation of SR-BI by PDZK1 seems to be hampered in hepatocyte cell culture since PDZK1, like canalicular markers, is altered in the transition process occurring from liver to primary hepatocytes (Tsukamoto et al. 2013).

Another determinant of SR-BI localization, at least in vitro, is the cellular cholesterol status. It was reported that basolateral–apical transcytosis of SR-BI occurs upon cholesterol loading in the polarized hepatocyte cell model WIF-B (Harder et al. 2007). In addition, cholesterol depletion triggers basolateral–apical transcytosis of SR-BI in MDCK cells (Burgos et al. 2004). These studies were performed in cells that overexpress SR-BI, which may already impact SR-BI distribution. In a physiological model of metabolic disturbance of the liver, the fasting and refeeding situation, where a transient fatty liver status can be seen, the overall distribution of SR-BI was unchanged; however, some intracellular staining was apparent. This is consistent with previous data showing that the hepatic cholesterol content was increased by more than sixfold in mice fed an atherogenic diet, and SR-BI expression decreased without an obvious change in the cellular expression pattern (Niemeier et al. 2009). Furthermore, in estrogen-treated rats, a model in which hepatic cholesterol is increased, there was no obvious alteration in SR-BI distribution (Stangl et al. 2002). When canalicular membranes of estrogen-treated rats were prepared, a similar SR-BI distribution pattern was found as in untreated rats (H. Stangl, unpublished data).

Numerous in vivo studies, which aimed at investigating the contribution of SR-BI to biliary cholesterol secretion, exploited genetic modification of SR-BI (Kozarsky et al. 1997; Mardones et al. 2001; Ji et al. 1999; Wiersma et al. 2009). Hepatic SR-BI overexpression increased biliary cholesterol secretion in mice; conversely, biliary cholesterol secretion was decreased in SR-BI knockout mice (Wiersma et al. 2009; Mardones et al. 2001). These are convincing observations, since the majority of cholesterol that is destined for secretion into the bile derives from selective cholesteryl ester uptake from HDL via SR-BI. It was previously shown that selective cholesteryl ester uptake from HDL into hepatocytes was blocked in SR-BI knockout mice (Out et al. 2004), emphasizing the important role of SR-BI at the sinusoidal membrane. However, we and others (Kozarsky et al. 1997; Ikemoto et al. 2000; Sehayek et al. 2003) detected SR-BI at the canalicular membrane in rodents and humans. The question remains to which extent SR-BI participates in apical cholesterol secretion or whether it has other functions at the apical membrane.

Despite fundamental differences in lipoprotein metabolism between rodents and humans, SR-BI loss of function has comparable effects at least on HDL cholesterol levels. Humans with SR-BI mutations (Brunham et al. 2011) as well as SR-BI knockout mice (Covey et al. 2003) display increased plasma HDL cholesterol levels, indicating that hepatic SR-BI expression in humans is as important as in mouse models. Our findings on SR-BI localization in the liver provide evidence for a common distribution pattern in rodents and humans.

In summary, two important observations arise from the present work. First, in vitro cultures of hepatocytes such as HepG2 cells show, among other limitations, considerable differences in the cellular localization of SR-BI compared to the in vivo situation. However, 3D human liver microtissues maintain a SR-BI distribution pattern that is similar to human and mouse liver. Thus, in vitro results studying SR-BI expression, especially those exploiting SR-BI loss or gain of function, should be evaluated with care. Second, the vast majority of SR-BI protein in hepatocytes of rodent and human liver resides at the sinusoidal membrane; however, a minor portion is clearly detected at the canalicular membrane.

## Electronic supplementary material

Below is the link to the electronic supplementary material.

Characterization of BC-like structures in HepG2 cells. HepG2 cells were cultivated under standard conditions to form monolayers. Immunofluorescence staining for MDR1 was performed, followed by phalloidin staining and confocal microscopy. Bar = 30 µm; bar for inset = 6 µm (EPS 513 kb)

Immunohistological analysis of SR-BI localization in HepG2, Huh7, and 3D human liver microtissues. **a-b**, HepG2 (**a**) and Huh7 (**b**) microtissues were grown in hanging drops, formalin fixed, paraffin embedded, sectioned, and stained for SR-BI. **c**, Immunofluorescence analysis of SR-BI in sections of microtissues generated from primary human hepatocytes and liver-derived non-parenchymal cells. The microtissues were cultured for 7, 14, 21, and 28 days. Note that the localization of SR-BI on the surface of the primary hepatocytes was preserved over 4 weeks in culture. Bar = 50 µm; bar for inset = 25 µm. Blue = DAPI (EPS 1260 kb)

Serial z-stack images of HDL-derived cholesterol delivery to BC-like structures. HepG2 cells were incubated with 50 µg/ml of HDL-BODIPY-cholesterol (green) for 15 min. Afterward, cells were fixed, and phalloidin staining (red) was performed. Cells were imaged using a confocal microscope. Two serial z-stack images are shown (compare with Fig. 1). Bar = 6 µm (EPS 648 kb)

## Abbreviations

3D: Three-dimensional
ABCB11: ATP-binding cassette transporter B11
BC-like: Bile canalicular-like
BP-C: BODIPY-cholesterol
CETP: Cholesteryl ester transfer protein
HDL: High-density lipoprotein
LDL: Low-density lipoprotein
LDLR: LDL receptor
MDR1: Multidrug resistance protein 1
MRP2: Multidrug resistance-associated protein 2
PDZK1: PDZ domain-containing protein 1
SR-BI: Scavenger receptor, class B, type I
VLDL: Very low-density lipoprotein
